# Mapping coexisting hotspots of multidimensional food market (in)accessibility and climate vulnerability

**DOI:** 10.1088/1748-9326/ad4400

**Published:** 2024-05-07

**Authors:** Gregory S Cooper, Bhavani Shankar

**Affiliations:** Institute for Sustainable Food and Department of Geography, University of Sheffield, Sheffield, United Kingdom

**Keywords:** food systems, markets, climate change, sustainability, India

## Abstract

With the increasing likelihood of agricultural production failures under a warmer global climate, the importance of markets in providing access to nutrient-dense foods (NDFs) through trade is predicted to grow. However, regions with relatively poor access to markets and supporting infrastructures (e.g. roads and storage facilities) are potentially ill-equipped to deal with both short-term hydrometeorological hazards such as droughts and floods, and longer-term shifts in agricultural productivity. Despite the increasing focus upon markets within academic and policymaking circles, a regional-scale assessment of these potentially coexisting hotspots of vulnerability has not been conducted. We conduct a two-stage geospatial analysis integrating three publicly available datasets across the Indian states of Bihar, Chhattisgarh, Jharkhand, and Odisha. Combining the 2011 national census with the new PMGSY-GeoSadak database, we conduct nearest neighbour analysis to measure multidimensional market inaccessibility by: (i) distance from a settlement to its nearest village, town or city with a market, (ii) distance from a settlement to its nearest major road, and (iii) distance from a settlement to its subdistrict headquarters. We then correlate these measures with India’s only district-wise assessment of climate vulnerability to identify hotspots of market inaccessibility and climate hazards. We find that the three market access measures are spatially autocorrelated and positively interrelated at the settlement (*n* = 129 555) and district (*n* = 107) levels, meaning that settlements located further from their nearest market tend to experience poorer road connectivity and access to the subdistrict economic hub. Approximately 18.5-million people live in districts with relatively high climate vulnerability and relatively high and multidimensional market inaccessibility. Hotspots of coexisting vulnerabilities are also disproportionately populated by ‘Schedule Castes and Schedule Tribes’ (SC/ST) communities. The identification of coexisting hotspots has important implications for the development of equitable and resilient markets that bolster NDF access for climate vulnerable and nutritionally insecure populations.

## 1 Introduction

Attributed to 3.9 million deaths worldwide in 2017 (WHO 2023), inadequate consumption of nutrient-dense foods (NDFs) such as fruits and vegetables (F&V) has been linked to higher rates of non-communicable diseases (Holt 2010), all-cause mortality (Aune *et al* 2017), and poorer levels of mental health (Głąbska *et al* 2020). Despite this, strengthening consumption of NDFs amongst nutritionally vulnerable populations remains a wicked problem—fraught with deep-rooted socioeconomic challenges and an absence of simple solutions.

Food markets supply the majority of NDFs consumed (e.g. fruits and vegetables and animal-source foods) in low- and middle-income countries (LMICs), and thus play an important role in enabling healthy diets (Zanello *et al* 2019, Matita *et al* 2021). While agriculture remains the predominant source of employment in rural regions of LMICs, and NDFs can be provisioned by farmer’s own production, most small farmers are also market-dependent for their food consumption for at least part of the year (e.g. during lean seasons). Additionally, increasing urbanisation and diversification of rural livelihoods beyond agriculture implies growing reliance on markets (de Bruin *et al* 2021), whilst the landless poor are often entirely market-dependent for their NDF consumption.

In a global climate that is warmer and more erratic (e.g. more extreme rainfall events), agricultural failures may reduce the reliability of food production (Caparas *et al* 2021), correspondingly increasing the importance of food traded between markets. Problematically, the implications of climate change for food systems extend beyond the farmgate to the current and future capacities of food markets to act as accessible and equitable sources of NDFs, with regions of low market density triple exposed to (a) reduced self-sufficiency in terms of food production, (b) reduced abilities to generate revenues from the sale of agricultural products at markets, and (c) relatively limited capacities to utilise markets to import food during local production failures.

Given its multidimensional nature, the concept of market access has different meanings in different contexts (Chamberlin and Jayne 2013, Nandi *et al* 2021). For example, villages in northern India located within walking distance of a ‘weekly market’ (i.e. generally open 1–3 d per week) may have good market access in terms of distance, travel times and costs. However, the importance of proximity might be reduced where affordable transportation networks enable rural communities to reach their nearest market or town—where permanent shops tend to operate every day and sell a diverse NDF range (Travasso *et al* 2023). Critically, these different dimensions of market access are vulnerable to the impacts of climate change. For instance, flooding from increasingly frequent and intense hydrometeorological hazards may isolate remote markets and villages from the wider transport, freshwater and energy networks. Even when market sites are physically accessible, the underdeveloped nature of facilities can undercut NDF availability and affordability (IFPRI 2022). For instance, the 30%–40% of agricultural production that is wasted each year in India is commonly attributed to the sparsity of climate sensitive facilities such as cold rooms, raised platforms and overhead canopies, which are particularly important during summer heat and monsoon rainfalls (Cooper and Shankar 2022, Travasso *et al* 2023). Regarding the functioning of markets, de Lima *et al* (2021) predict 30%–50% declines in agricultural labour capacity across sub-Saharan Africa and Southeast Asia under warming scenarios of 3 °C above the 1986–2005 baseline. Such issues are particularly corrosive to market access in rural areas, where consumers with restricted mobility and income opportunities (e.g. seasonal labour) may depend upon a single weekly market.

To date, whilst studies have uncovered important insights into how food system actors and functions can resist and recover from systemic stresses (Schipanski *et al* 2016, Béné *et al* 2023), this study is motivated by recent reviews which highlight how research has predominantly focused on (i) the vulnerability of *agricultural production* to climate change, as opposed to the markets and allied infrastructures underpinning the trade of food, and (ii) staple food items such as wheat and rice, as opposed to NDFs critical to tackling malnutrition (Meyer 2020, Davis *et al* 2021). In a recent attempt to bridge these gaps, Scharadin *et al* (2023) explored the relationships between extreme weather and the retail food environment in the United States, finding that relatively climate vulnerable counties are associated with both lower levels of retail outlet availability and disproportionately high proportions of low-income populations. In India, where an estimated 1-billion people cannot reliably afford a healthy diet (FAO *et al* 2023), and where socioeconomic disadvantages such as caste can widen food system inequalities (Choudhury *et al* 2020), plausible differences in market access and climate vulnerability have potentially deleterious implications for food and nutrition security.

Therefore, this study aims to (1) identify and quantify hotspots of multidimensional market (in)access across four nutritionally vulnerable states of India, including the magnitudes of inequality and the interrelations between the different dimensions of market access, and (2) explore the coexistence of hotspots of climate change vulnerability and market inaccessibility to identify priority regions facing multidimensional stresses. This analysis utilises three publicly available datasets (section 2.1), with nearest neighbour analysis first used to define distances from census villages to nearest markets, major roads and urban centres, before exploring the association between market access and India’s only district-level measure of climate vulnerability (Mohanty and Wadhawan 2021). In addition to enhancing understanding into the implications of climate change for the critical food system elements downstream of the farmgate, this study also has salient implications for policymakers. With the Government of India currently planning to upgrade the facilities of 22 000 existing rural markets nationwide, whilst concurrently increasing the density of wholesale markets from one every 463 square kilometres to one every 80 square kilometres (Government of India 2019, 2020), this analysis provides (a) insights into the types of improvements which should be prioritised in different locations, such as the development of new markets versus the addition of climate-resilient infrastructure in existing markets, and (b) a horizon scan of the implications of developing new markets where they currently do not exist.

## 2 Methods

### 2.1 Datasets and data preparation

We focus our analysis on the contiguous states of Bihar, Chhattisgarh, Jharkhand, and Odisha—four states where agri-food systems form the predominant livelihood source (Thomas 2023), but state-wise scores of child malnutrition and multidimensional poverty consistently rank within the highest quartiles (NITI Aayog 2023). Utilising three newly compiled national datasets (table 1), all geospatial analysis was conducted in the software QGIS (v3.10), with outputs exported in CSV format for analysis in R (R Core Team 2020).

The Socioeconomic High-resolution Rural-Urban Geographic Data Platform for India (‘SHRUG’) has digitised over a dozen national sociodemographic and environmental datasets (Asher *et al* 2019). Using SHRUG’s common census tract identifier ‘*shrid*’, the ‘PC11 Village Polygons’ dataset containing the spatial boundaries of all 2011 national census tracts was merged with the demographic datasets ‘2011 Population Census Village Directory’ and ‘2011 Population Census Town Directory’ (i.e. containing population, sociodemographic and infrastructure data). Villages, towns and cities (hereafter referred to as ‘settlements’) located within the four study states were then subset (*N* = 129 555), and the geographical centre of each settlement was identified using the QGIS ‘Centroids’ tool.

Road locations for each state were obtained from their respective District Rural Road Plan (DRRP) datasets, via the Government of India’s GeoSadak portal released to the public in early 2022 (table 1). In line with Snapp and Fisher (2015), Kissoly *et al* (2018) and Wudad *et al* (2021), we include only major paved roads, reflecting the importance of perennially passable roads in connecting producers and consumers to local markets. The QGIS filter was used to include roads classified as ‘National Highways’, ‘State Highways’, ‘Major District Roads’ and ‘Other District Roads’, thus omitting ‘Rural Roads (tracks)’ and ‘Other Rural Roads’.

The third national dataset is the district-wise climate change vulnerability index developed by Mohanty and Wadhawan (2021). Based on the climate vulnerability measure of IPCC (2012, 2012), the dataset is India’s first district-wise climate vulnerability assessment, developed by integrating data on hazard exposure (e.g. historical records of extreme hydrometeorological events), landscape sensitivity (e.g. land use, ground water and soil moisture), and adaptive capacity of the population and government (e.g. disaster risk management plans, gross district domestic product and population density). Therefore, whilst the exposure and sensitivity dimensions of the measure are relatively aligned with the biophysical determinants of agricultural production, the adaptive capacity dimension more closely reflects the demand for consumable food and the maintenance of functioning food distribution systems and markets—especially during climate hazards (Mohanty and Wadhawan 2021, table 1).

### 2.2 Geospatial measures of market access

**Figure 1:**
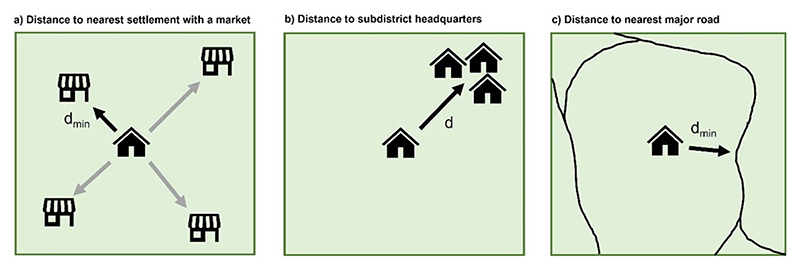
Graphical representation of the three geospatial measures of market access. Symbols: *d*—‘distance’, *d*_min_—minimum distance.

Multiple measures of market access have emerged over the past decade, centred around market distance, travel time, and the cost to reach the nearest market (Nandi *et al* 2021). Traditionally, such studies are based on surveys capturing hundreds or thousands of households; the analysis here defines three metrics of market access across 129 555 settlements (figure 1):

- *Distance from each settlement to the nearest settlement with a market:* The QGIS ‘distance to nearest hub (points)’ tool calculates the distance from each census settlement to the nearest settlement with either a ‘regular market’ or a ‘weekly haat’ (i.e. variables ‘pc11_vd_mrkt’ and ‘pc11_vd_wkl_haat’, respectively, in the SHRUG ‘2011 Population Census Village Directory’). Without the data or computational capacity to route all 129 555 settlements through the road network, this measure defines the minimum possible Euclidean distance between settlements. Settlements with either a regular market or weekly haat (*small market* in Hindi) are assigned 0 km, whilst all urban settlements featured in the SHRUG ‘2011 Population Census Town Directory’ are assumed to have a market. Acknowledging that consumers may cross state boundaries to access markets, settlements up to 10 km outside of the study states were also included.
- *Distance from each settlement to its subdistrict headquarters:* In order to save time and money through the bundling of activities, rural consumers may visit the nearest town when other household items (e.g. clothes and school items) are to be purchased alongside food (Kehoe *et al* 2019, Travasso *et al* 2023). This metric also aims to capture an element of permanence, with sources of NDFs in urban areas often open morning to evening daily, whilst stocking a richer diversity of NDFs compared to village markets. Distances to the subdistrict (i.e. block) headquarters for each census tract are available in the SHRUG ‘2011 Population Census Village Directory’ (i.e. variable ‘pc11_vd_subdistrict_hq_dist’).
- *Distance from each settlement to the nearest major road:* All-weather roads are critical to link producers, consumers and associated food trade to markets throughout the year (Rammelt and Leung 2017, Weatherspoon *et al* 2017). As per *Metric A*, the Euclidian distance from each settlement to the nearest major road is calculated using the QGIS tool ‘distance to nearest hub (points)’.

It is worth describing the spatial and temporal congruence of the datasets here. With the climate vulnerability values of Mohanty and Wadhawan (2021) only available at the district level, the settlement-wise market access measures described above are aggregated by averaging the values within each district, weighted by the 2011 census population of each settlement (i.e. populous settlements contribute proportionally more to the district averages). From here, district level market access measures were matched with the climate vulnerability figures using the ‘join variables by field value’ tool in QGIS to match district names. In line with the climate vulnerability index, the district boundaries of the 2011 national census are used (*n* = 113); however, the districts of Bilaspur, Korba and Janjgir-Champa in Chhattisgarh are excluded owing to data omissions in the SHRUG ‘2011 Population Census Village Directory’.

It is important to highlight that the datasets also have different temporal coverages: distances to the nearest settlement with a market, the subdistrict headquarters, and the district-wise climate vulnerability index are all derived from the 2011 census, whilst the measure of major road distance uses the continuously updated GeoSadak portal—meaning that the dataset may include roads built since the last national census in 2011. In terms of alternative datasets, whilst the ‘Agricultural Facilities’ data of the GeoSadak portal presents another source of market locations, the spatial coverage of the dataset is relatively limited; for instance, GeoSadak identifies fewer than 200 markets in Bihar, whilst the census identifies 14 496 settlements with markets. Therefore, to assess the implications of these different temporal horizons and associated uncertainties on the confidence of our findings, we conduct sensitivity analyses on: (i) the strengths of associations between district-level measures of market access (supplementary materials D1), and (ii) the spatial patterns of coexisting hotpots of market inaccessibility and climate vulnerability (supplementary materials D2).

## 3 Results

### 3.1 Hotspots of market inaccessibility

We explore the extent to which the different measures of market access are interrelated and clustered across space, before quantifying the population living with different levels of multidimensional market inaccessibility.

**Figure 2:**
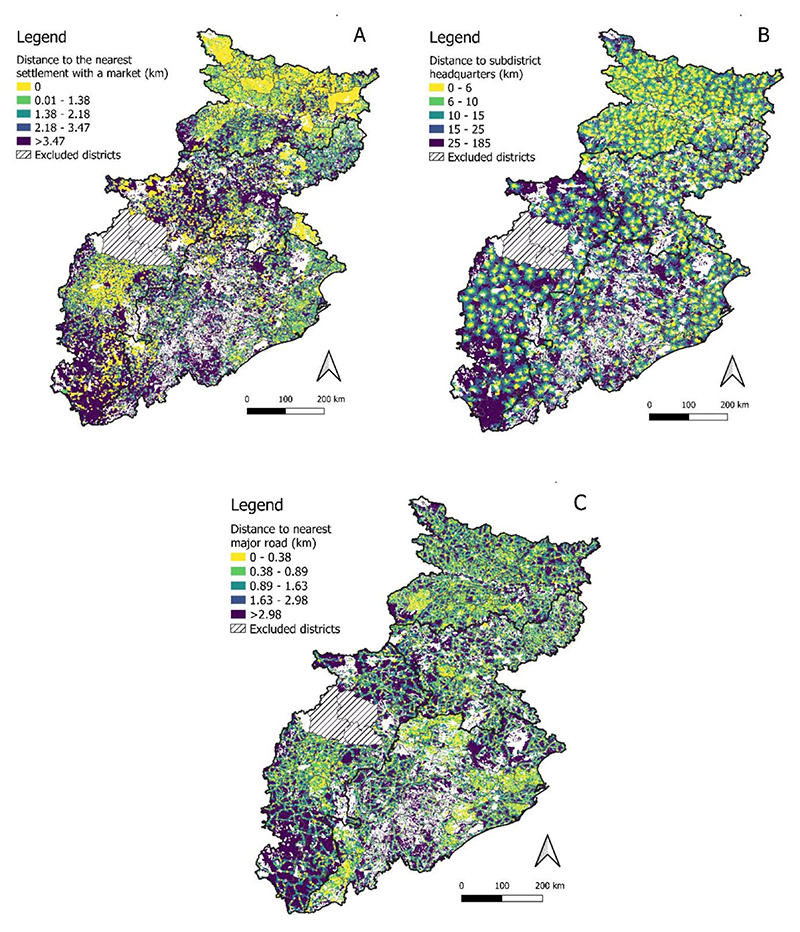
The three market access metrics plotted across 129 555 census tracts in Bihar, Jharkhand, Chhattisgarh and Odisha (A) distance to nearest settlement with a market, (B) distance to the subdistrict headquarters, and (C) distance to the nearest major road. See Supplementary Material B for the state-wise summary statistics.

**Figure 3:**
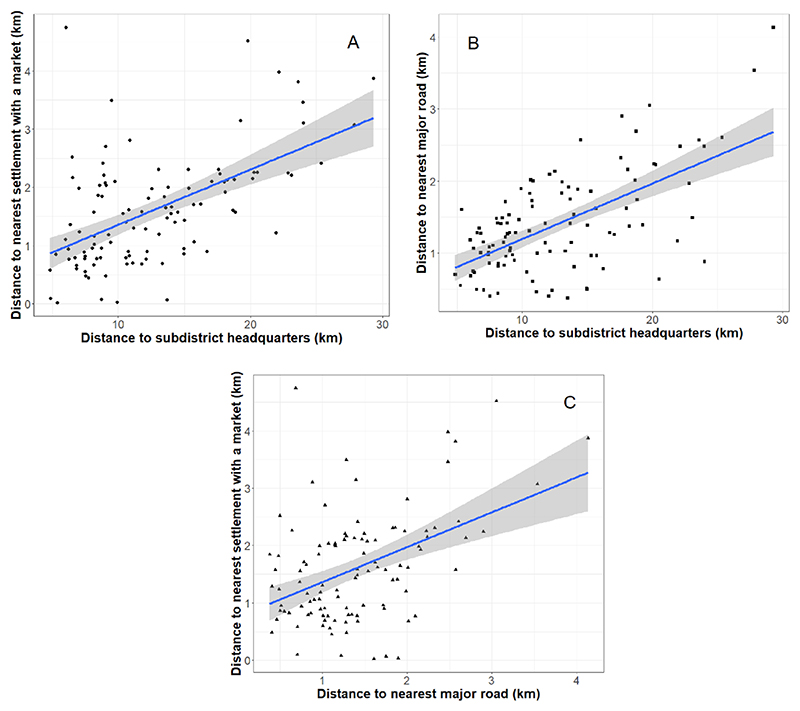
District-wise bivariate relationships between the three market access metrics. Corresponding correlation and linear regression outcomes (A) *R* = 0.559, *R*^2^ = 0.306, *p <* 0.001, *df* = 105, (B) *R* = 0.616, *R*^2^ = 0.374, *p <* 0.001, *df* = 105, and (C) *R* = 0.447, *R*^2^ = 0.192, *p <* 0.001, *df* = 105. The grey highlights represent the 95% confidence interval of the linear regression models.

All three market measures (figure 2) exhibit statistically significant (i.e. *p <* 0.001) clustering at the settlement level, as defined by Moran’s I statistic (Moran 1950). This means that settlements located far from markets are located relatively close to settlements also far from markets, and significant spatial autocorrelations pertain for distances to the sub-district headquarter and nearest major road (supplementary material A). In addition to the associations with space, all three market indicators are positively correlated at the district (figure 3) and settlement scales (supplementary material C). Market inaccessibility at the regional scale is therefore multi-dimensional and compounding, with settlements located relatively far from their nearest weekly or regular market found to be relatively distant from their subdistrict headquarters, where permanent retail outlets commonly provide a reliable source of NDFs for consumers (Travasso *et al* 2023). Such settlements are also found to be located relatively far from the nearest major road, which further limits convenient and cost-effective access to markets. Therefore, in general, the lack of market access in one dimension (e.g. market distance) cannot be compensated by market access in a different dimension (e.g. major road distance). These associations remain robust to potential uncertainties in the data and geospatial analysis approaches (supplementary material D1).

Through Census of India 2011 data, it is possible to quantify and characterise the populations with extreme multidimensional market access (table 2). For instance, of the total population of *~*198 million, approximately 102 million (51.4%) live in settlements with a weekly haat or regular market (table 2). Of these, 33.3 million (16.8%) live in settlements situated within the best performing deciles across all three metrics (i.e. a settlement with a market, within 0.178 km of major road *and* within 3 km of the subdistrict headquarters). At the opposite end of the scale, 8.28 million (4.2%) live in settlements located at least 4.88 km and 1 million (0.5%) live in settlements located 10 km from the nearest settlement with a market, respectively. Combining these dimensions, over 1 million people live in settlements located at least 4.88 km from the nearest settlement with a market, whilst simultaneously beyond 4.42 km from the nearest major road and more than 35 km from the subdistrict headquarters.

The census data also uncovers potential systemic inequalities, with the populations at the extreme ends of market access differentiated by caste composition. For all three metrics and their combinations (table 2), settlements in the best performing deciles average SC/ST proportions (i.e. the most disadvantaged caste groups) beneath the 28.2% average for the region, whilst settlements in the worst performing deciles average higher. In turn, the proportion of the population that is SC/ST located in settlements in the worst performing deciles (76.2%) across all three metrics is more than four times the equivalent percentage for the settlements falling in the best performing deciles (18.1%).

### 3.2 Coexisting hotspots of market inaccessibility and climate vulnerability

Multiple dimensions of market access have been found to cluster and positively correlate at both the settlement and district levels, whilst settlements with the poorest multidimensional market access are disproportionately home to populations from disadvantaged castes. Here, market inaccessibility is combined with the climate vulnerability scores of Mohanty and Wadhawan (2021) to identify hotspots of coexisting vulnerabilities. Districts scoring higher than the mean climate vulnerability score (=0.309, weighted by district population) for the 107 districts are classified as relatively climate vulnerable, whilst for each of the three market access metrics, districts are relatively vulnerable to that component of market access if their value exceeds the weighted average for all 107 districts in the region. Districts then become multidimensionally vulnerable if more than one access metric exceeds the regional average.

**Figure 4:**
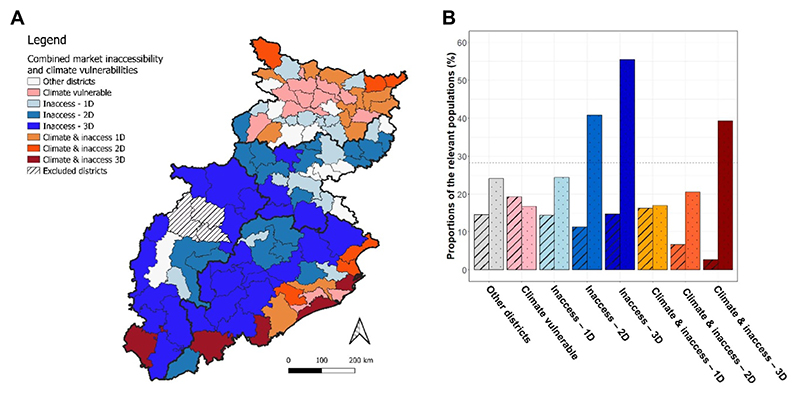
(A) Composite map combining the district-wise climate vulnerability scores of Mohanty and Wadhawan (2021) with the number of relative market inaccessibility dimensions (i.e. 1D, 2D or 3D); (B) bar-chart plotting the percentage of the total regional population within each vulnerability classification (dashed bars) and the proportion of each classification’s population that is classified as SC/ST (dotted bars). The horizontal reference line is the percentage of the total regional population that is SC/ST (28.2%).

Districts can be classified into distinct groups along these two vulnerability dimensions (figure 4(A)). First, located predominantly in eastern Jharkhand (e.g. Bokaro and Dhanbad) and south of the River Ganges in Bihar (e.g. Gaya and Nawada), 11 districts are classified as neither vulnerable to climate extremes nor market inaccessibility. Second, 44 districts experience multidimensional market inaccessibility, but relatively low rates of climate vulnerability. With a combined total population of over 28 million, major hotspots are concentrated in the belt running from north to south-central Odisha, south Jharkhand, and north and south Chhattisgarh.

Third, 23 districts predominantly clustered in coastal Odisha and north-eastern Bihar experience relatively high climate vulnerability coupled with at least one dimension of relative market inaccessibility (figure 4(A)). Of these, 11 districts, home to approximately 18.5-million people in 2011, are both relatively climate vulnerable and score relatively poorly across two or more market metrics, with five of these districts (i.e. Bijapur in Chhattisgarh, and Koraput, Gajapati, Puri, and Kendrapara in Odisha) scoring relatively poorly across all three market access measures (5.30-million people). Reflecting the trend indicated in section 3.1, disadvantaged castes constitute 39.3% of the total population in these most multidimensionally vulnerable districts, which is 39.4% above the average for the region. Disadvantaged castes are also disproportionately represented in the districts with multidimensional market inaccessibility (but low climate vulnerability), constituting 40.8% and 55.4% of the total populations in the two-dimensional and three-dimensional classifications, respectively (figure 4(B)).

## 4 Discussion

### 4.1 Study implications

This study finds evidence to support the multidimensional and compounding nature of market inaccessibility: settlements located further from weekly markets also experience lower access to permanent sources at the subdistrict headquarters, and poorer major road connectivity required to reliably reach alternative markets. These associations are found to be robust at different scales of analyses (supplementary material C) and to the uncertainties underlying the data (supplementary material D).

These findings have implications for food and rural development policies in our study region, especially in the context of climate change. Judicious, cost-effective investments in road and/or market infrastructure are critical, especially in the areas identified in this research as lacking accessibility along multiple dimensions. As India urbanizes and structural transformation of the economy continues apace, it is important that rural areas can efficiently release surplus labour for the growing urban sector. It is also important that agricultural productivity and commercialization grows in rural areas to enable improved rural incomes and meet rising food demand in the face of a shrinking agriculture sector. Road and market infrastructural development has a key role to play in enhancing labour mobility, agricultural productivity, and commercialisation (Barrett 2008, Stifel *et al* 2016). Asher and Novosad (2016) find that lowering economic remoteness by connecting villages to roads in India has had a significant influence on reallocating labour from agriculture into wage labour, while Barrett *et al* (2022) note that reducing the remoteness of farmers from markets has a greater impact on agricultural commercialization than trade or macroeconomic policies. Moreover, Shamdasani (2021) finds that households in previously remote communities in India increased their uptake of new technologies and proportion of production sold to market following improved road connectivity and diversification into high-return crops.

A stream of recent research has also shown that reducing economic remoteness to markets and improving the quality of local markets are important for improving dietary and child growth outcomes (Hirvonen and Hoddinott 2017, Sibhatu and Qaim 2017, Headey *et al* 2019). This happens through multiple pathways, namely improved incomes via specialization, productivity and commercialization gains, reduced local food prices and access to greater food diversity. Furthermore, recent research has also shown that nutrition-sensitive social protection programmes such as cash transfers can falter when food markets function poorly. For example, Filmer *et al* (2023) find that a cash transfer programme in the Philippines worsened nutrition outcomes for non-beneficiaries in remote villages, by raising the demand for perishable foods without a corresponding ability to improve supply through well-functioning markets.

Therefore, the findings of our study underscore the urgent need to strengthen market and road infra-structure in districts with coexisting hotspots of climate vulnerability and market inaccessibility. Food markets have been shown to attenuate the adverse effects of weather shocks on nutrition (Mulmi *et al* 2016, Darrouzet-Nardi and Masters 2017, Dietrich and Schmerzeck 2019), with Shively (2017) calling for greater investment in market infrastructure in rural Nepal, finding that households more isolated from local markets suffer more pronounced nutritional impacts from weather shocks. Moreover, in Odisha, Spiker *et al* (2023) find that growth in agricultural productivity alone without associated improvements in market, transport and storage infrastructure may actually reduce NDF availability through heightened food loss rates. Simultaneously experiencing the lowest road densities, greatest distances between settlements and markets, and the highest proportions of populations belonging to disadvantaged castes, the 11 districts identified here are the least well-positioned to build climate resilience through the market-based trade of NDFs.

This study also highlights an important dimension of socioeconomic inequality in the Indian food system: districts which perform poorly in all three market accessibility measures *and* climate vulnerability are disproportionately populated by SC/ST communities. Disentangling causation between the geographical distributions of caste and market access (i.e. disadvantaged castes located in remote or forested regions versus regions receiving less development attention due to caste distributions) is beyond the scope of this paper. Nevertheless, the extent to which regions with poorest market access are disproportionately populated by the most disadvantaged castes is striking, and consistent with broader patterns of caste-based inequalities, including differential rates of F&V consumption (Choudhury *et al* 2021), dependence upon wage labour (Mosse 2018), and land ownership (Khan *et al* 2021). Market inaccessibility in such districts is potentially compounded by lower rates of asset ownership amongst disadvantaged castes, extending to house-hold income and the ownership of motor vehicles (Tagade and Thorat 2020). Further afield, these market inequalities are consistent with those uncovered in the United States by Scharadin *et al* (2023), whereby climate vulnerable localities with poor quality food environments are disproportionately populated by minority ethnic groups and low-income groups.

While this study has highlighted the importance of road and market infrastructure investments and indicated priority geographies in the study region, it is important that such investments are targeted, locally appropriate, equitable, developed using participatory approaches, and cost-effective. Benefits from investment may accrue from a variety of pathways and may strengthen over time, including via improvements in agricultural productivity and commercialization, labour mobility, and dietary improvements. Yet, infrastructure is not a panacea, and both initial investment and continuous subsidisation can threaten viability. Also, with construction being a primary (i.e. clearance for roads) and secondary (i.e. economic activities resulting from roads) driver of deforestation (Dalin and Rodríguez-Iturbe 2016, Seekell *et al* 2018), development should prioritise existing roads to limit furthering environmental degradation. Such sensitivity is pertinent in states such as Chhattisgarh, Jharkhand and Odisha, where indigenous communities often derive food and livelihoods from forest ecosystems (Ghosh-Jerath *et al* 2021).

### 4.2 Study limitations and future directions

This study is not without limitations. Census data on market locations only indicates the binary presence of a regular market and/or a weekly haat within a settlement, as opposed to the number of markets, their size, periodicity, or existing infrastructure. Consequently, overall market density is likely underestimated, particularly in urban areas, where multiple retail outlets often exist. As outlined in section 2.2, the datasets have different temporal coverages, with the distances to the nearest settlement with a market, the subdistrict headquarters, and the district-wise climate vulnerability index all derived from the 2011 census (now more than a decade old), whilst the measure of major road distance is derived from the ‘live’ GeoSadak portal. Positively, the sensitivity analysis conducted to ascertain the implications of these uncertainties found that the strengths of the associations between market access measures and the spatial patterns of vulnerability hotspots remain robust to a range of potential error magnitudes in the datasets (supplementary materials D).

It should also be noted that the climate vulnerability index of Mohanty and Wadhawan (2021) was not purposefully constructed to measure food system vulnerability. Instead, the measure aims to quantitatively compare the general vulnerability of India’s districts, based around historical exposure to extreme climatic events, the sensitivity of the landscape to such events, and the socioeconomic capacities of districts to prepare and adapt. Therefore, whilst the sensitivity and exposure dimensions align with the vulnerability of agricultural production, and adaptive capacity more closely with food distribution and consumption, future research may seek to disaggregate these dimensions to further tailor policy recommendations to specific vulnerability combinations. For example, recommendations for relatively exposed districts that also happen to be relatively adaptive might differ from districts with the opposite configuration.

Moreover, the fact that the index is based upon historical records of extreme weather, rather than future climate projections, has important implications for this study. With climate change projected to alter the magnitude and frequency of hydrometeorological events, this study assumes that relatively unexposed districts will remain so, and the comparative ranking of district climate vulnerabilities will persist. Moreover, this study represents a static snapshot in time, thus potentially underplaying the spatiotemporal dynamics that exist between climate, agriculture and associated policymaking at multiple scales (e.g. domestic and international). For instance, in response to the Russia-Ukraine war, Chai *et al* (2024) forecast an 8.4 mega-hectare expansion in cropland globally (of which 0.3 MHa is expected to occur in India), triggered by a cascading effect whereby nations increase domestic production and strengthen existing trade partnerships to compensate for losses due to the conflict. Therefore, whilst cropland expansion is projected to occur mainly in India’s northwestern states (e.g. Punjab and Rajasthan), the analysis conducted here does not account for any changes in cropland or market density in our study sites resulting from cross-boundary global cascades triggered by conflict or climate change. In the case of India, such cross-boundary cascades may be triggered from relatively climate vulnerable states and/or districts (e.g. coastal Odisha), with implications for the agriculture, livelihoods, and the environment of neighbouring regions (e.g. inland Odisha). Therefore, while this current analysis may help to inform policies of cropland expansion, for example, by assessing the climate vulnerability and the extent to which candidate locations have the infrastructure to support changes in production, future research may seek to include downscaled dynamic climate projections and explore subsequent scenarios of agricultural expansions or contraction to understand how vulnerability hotspots may change in location and size in future.

Given the scope and spatial scale of this study, only three out of the possible myriad of market access metrics were analysed (Chamberlin and Jayne 2013, Nandi *et al* 2021). As such, dimensions other than physical distance, such as time-use or financial cost, have been omitted. Similarly, given data availability restrictions, this study focused only on the physical nature of market access, thus excluding elements of the personal food environment which can moderate individuals’ interactions with markets, such as food hygiene, perceptions of security, and personal mobility (Turner *et al* 2018). Moving forward, this study could be complemented by a smaller-scale mixed-methods analysis seeking to validate the regional findings, with traditional quantitative survey approaches (e.g. household surveys) exploring how market distances influence costs and time-use, and qualitative approaches (e.g. interviews) establishing the importance of distance relative to the personal food environment in market-related decisions.

## 5 Conclusion

Through the geospatial analysis of three recently compiled national level datasets, this study has identified coexisting hotspots of market inaccessibility and climate vulnerability across four states of India. The three measures of market access are found to be spatially autocorrelated and interrelated, with localities situated further from markets also generally located further from major roads and subdistrict headquarters. With approximately 18.5 million people living in districts with the highest relative levels of climate vulnerability *and* multidimensional market inaccessibility, climate resilience must be prioritised in the Indian government’s drive to upgrade over 20 000 rural markets, including a mix of long and short supply chains, cold storage facilities and raised platforms. However, these investments are not a silver bullet, and to be sustainable in the long-run and ensure that nobody is left behind, such initiatives must be cost effective (e.g. to bring both policymakers and private investors on board) and integrated with the traditional pathway of structural transformation which seeks to enhance urban-rural connectivity, livelihood opportunities and rural incomes.

## Supplementary Material

Supplementary material

## Figures and Tables

**Table 1:** Description of the datasets and corresponding variables analysed in this study. Acronyms: SHRUG—socioeconomic high-resolution rural-urban geographic platform for India; PMGSY—Pradhan Mantri Gram Sadak Yojana (Prime Minister’s Village Road Scheme); SC/ST — Scheduled Castes/Scheduled Tribes.

Dataset	Variable	Source
Census of India, 2011	Census tract locations (i.e. villages, towns and cities)	SHRUG—‘PC11 Village Polygons’ data
	Total population of villages	SHRUG—‘2011 Population Census Village Directory’
	Total population of towns and cities	SHRUG—‘2011 Population Census Town Directory’
	SC/ST population of villages	SHRUG—‘2011 Population Census Village Directory’
	SC/ST population of towns and cities	SHRUG—‘2011 Population Census Town Directory’
	Distance to subdistrict headquarters	SHRUG—‘2011 Population Census Village Directory’
	Villages with ‘regular market’ and/or ‘weekly haat’	SHRUG—‘2011 Population Census Village Directory’
PMGSY GeoSadak open data	Locations of major roads	District Rural Road Plan datasets for the four states
India’s ‘Climate Change Vulnerability Index’	District-wise climate vulnerability score	Centre for energy, environment and water (CEEW)
Vegetation continuous fields (VCF)	Village and district-wise measures of land proportion covered by vegetation (Supplementary Material E).	SHRUG—‘Forest cover’

**Table 2:** Characteristics of the populations living in settlements located within the lowest (i.e. best access) and highest (i.e. worst access) deciles of the three metrics of market access. Rows labelled with ‘*’ combine two market access metrics, whilst rows labelled with ‘†’ combine all three. Note: rows are not mutually exclusive; for example, a settlement in the highest decile of market distance may also be in the highest decile of major road distance. SC/ST—Scheduled Castes and Scheduled Tribes.

		Best performing decile(s)				Worst performing decile(s)		
Market access metric *(distance to the nearest…)*	Median distance (km)	Maximum distance (km)	Population (million)	SC/ST (%)		Minimum distance (km)	Population (million)	SC/ST (%)
Market	1.75	0	101.7	22.3		4.88	8.28	55.9
Major road	1.22	0.178	50.8	21.4		4.42	9.18	48.5
Subdistrict HQ	12	3	52.9	21.1		35	7.78	51.5
Market & major road*	As above	As above	40.8	19.1		As above	1.37	72.0
Market & subdistrict HQ*	As above	As above	41.2	18.7		As above	1.04	76.2
Major road & subdistrict HQ*	As above	As above	35.1	18.5		As above	7.77	51.5
All three metrics^†^	As above	As above	33.3	18.1		As above	1.04	76.2

## Data Availability

The data that support the findings of this study are openly available from the sources detailed in table 1. No new data were created or analysed in this study.
