# Supplementary material for: Mapping coexisting hotspots of multidimensional food market (in)accessibility and climate vulnerability
Source: Environ Res Lett. Author manuscript; Available in PMC 2024 Jul 31. (PMC7616319; doi:10.1088/1748-9326/ad4400)
Supplement: Supplementary material [file EMS197741-supplement-Supplementary_material.pdf]

ENVIRONMENTAL RESEARCH
LETTERS

Supplementary material for this article is available online

© 2024 The Author(s). Published by IOP Publishing Ltd
